# Combined Unilateral Hemilaminectomy and Thoracoscopic Resection of the Dumbbell-Shaped Thoracic Neurinoma: A Case Report

**DOI:** 10.1155/2012/517563

**Published:** 2012-08-30

**Authors:** Toshihide Tanaka, Naoki Kato, Ken Aoki, Aya Nakamura, Mitsuyoshi Watanabe, Satoru Tochigi, Hideki Marushima, Tadashi Akiba, Yuzuru Hasegawa, Toshiaki Abe

**Affiliations:** ^1^Department of Neurosurgery, Jikei University School of Medicine, Kashiwa Hospital, 163-1 Kashiwa-shita, Chiba, Kashiwa 277-8567, Japan; ^2^Department of Surgery, Jikei University School of Medicine Kashiwa Hospital, Kashiwa Hospital, 163-1 Kashiwa-shita, Chiba, Kashiwa 277-8567, Japan; ^3^Department of Neurosurgery, Jikei University School of Medicine, Tokyo 105-8461, Japan

## Abstract

A 41-year-old woman complained of chest pain when coughing. Computed tomography and magnetic resonance imaging disclosed a homogenously enhanced tumor occupying the spinal canal at the Th7 level and extending into the right paravertebral space through the intervertebral foramen between Th7 and Th8. The tumor was successfully removed via a posterolateral approach using unilateral hemilaminectomy followed by thoracoscopic surgery. Since the tumor had a dumbbell shape, a combined approach was considered essential. The histological diagnosis was a thoracic neurinoma. Combined hemilaminectomy and thoracoscopic surgery may be a good alternative for the management of thoracic dumbbell-shaped tumors.

## 1. Introduction

Dumbbell-shaped tumors account for 6 to 14% of all spinal tumors [1, 2], and approximately 35% of all neurinomas of the spinal canal originate in the thoracic region [3]. The standard procedure for removing posterior mediastinal neurogenic dumbbell-shaped tumors is a two-stage, combined neurosurgical and thoracic approach with thoracotomy [4]. Most dumbbell-shaped tumors can be resected effectively with partial laminectomy or unilateral hemilaminectomy or unilateral facetectomy [5, 6]. However, if the extraforaminal tumor component is large, a combined posterolateral approach should be utilized. 

Recent reports have described the use of video-assisted thoracic surgery for the resection of dumbbell-shaped tumors [7–12]. Combined laminectomy and thoracoscopic surgery has been used to remove dumbbell-shaped tumors that have a large extraforaminal tumor component. Thoracoscopy may be used in place of thoracotomy to resect intrathoracic tumors, because its incisions are less traumatic than thoracotomy and result in less postoperative pain, improved shoulder function, and lower morbidity [9, 13].

This study describes a case of a dumbbell-shaped thoracic neurinoma successfully treated by combined unilateral hemilaminectomy for the intracanalicular portion of the tumor and with thoracoscopic surgery for the extraforaminal, retropleural portion of the tumor.

## 2. Case Report

A 41-year-old woman complained of chest pain when coughing and was incidentally diagnosed with paravertebral tumor in the right posterior mediastinum. Neurological examination on admission showed no definite abnormalities. The patient had no history or cutaneous lesions suggesting neurofibromatosis. 

**Figure 1:**
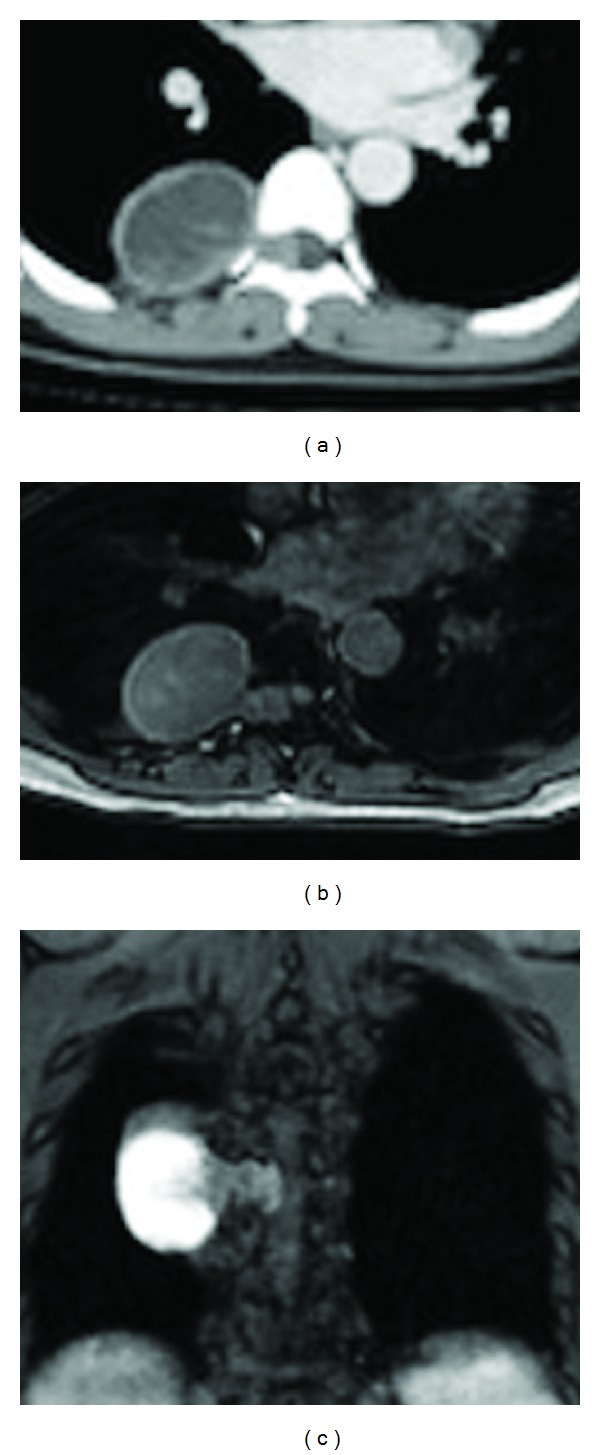
(a) Computed tomography (CT) scan showing a tumor occupying the spinal canal and extending into the paravertebral space through the right intervertebral foramen between Th7 and Th8. Axial (b) and coronal (c) T1-weighted magnetic resonance images with gadolinium enhancement, revealing a well-demarcated enhancing dumbbell-shaped tumor at Th7/8 on the right side. The tumor compresses the spinal cord anterolaterally towards the left side.

A computed tomography (CT) scan demonstrated a thoracic epidural tumor occupying the spinal canal and extending into the right paravertebral space through the intervertebral foramen between Th7 and Th8 (Figure 1(a)). Coronal magnetic resonance (MR) imaging showed a hyperintense tumor on the T2-weighted image at Th7 (Figure 1(b)). Axial MR imaging showed that the thoracic cord was markedly compressed on the right side and that there was cystic degeneration of the extracanalicular portion (Figure 1(c)). Based on these data, a preoperative diagnosis of thoracic dumbbell-shaped neurinoma was made. 

**Figure 2:**
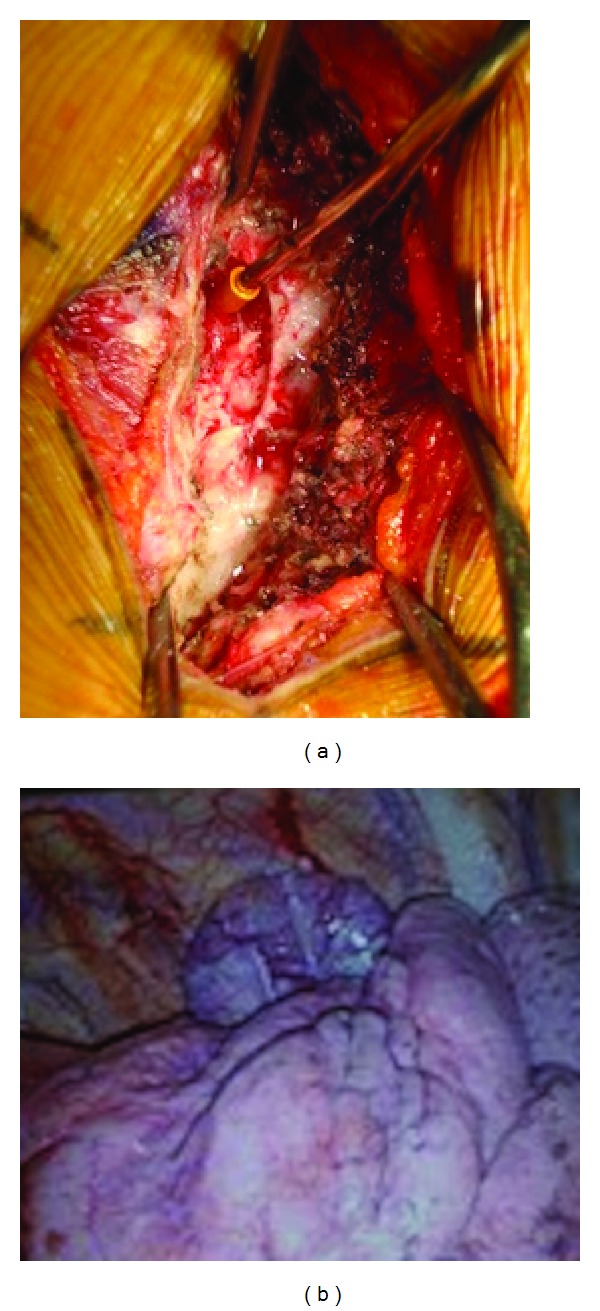
Intraoperative photographs demonstrating the intrathoracic (a) and intrapleural region (b). The extradural tumor was exposed by right unilateral hemilaminectomy (a). The decompressed tumor was identified, and the parietal pleura overlying the tumor were divided (b).

The intraspinal tumor component was removed first. The patient was intubated and anesthetized and placed in the prone position. Right unilateral hemilaminectomy with partial transversectomy of Th7 was performed. All ligamentous attachments were carefully stripped from the transverse process. The right posterior spinal arch from Th7 and Th8 was exposed. The round tumor was clearly seen following incision of the intertransverse ligament, bulging out between the Th7 and Th8 transverse process (Figure 2(a)). The tumor was located extradurally, and internal decompression was performed with minimum retraction to the dural sac wherever possible. The intra- and extracanalicular portions of the tumor were connected by a narrow stalk through the foramen. The intracanalicular portion of the tumor was carefully separated, and the fluid within the extracanalicular portion was drained.

The extraforaminal tumor was approached with right thoracoscopic surgery. The patient was placed in the left lateral decubitus position, and a Univent tracheal blocker tube was inserted to the right bronchus to enable left-lung ventilation. Stab wounds were made in the anterior axillary line at the third, fourth, sixth, and seventh intercostal space, and at the fourth intercostal space adjacent to the tumor. Once the tumor was identified, the parietal pleura overlaying the tumor were divided to allow the tumor to be dissected free from the vertebra (Figure 2(b)). The tumor was completely removed through the endoscope. Incisions were closed after reinflation of the lung under direct visualization. 

**Figure 3:**
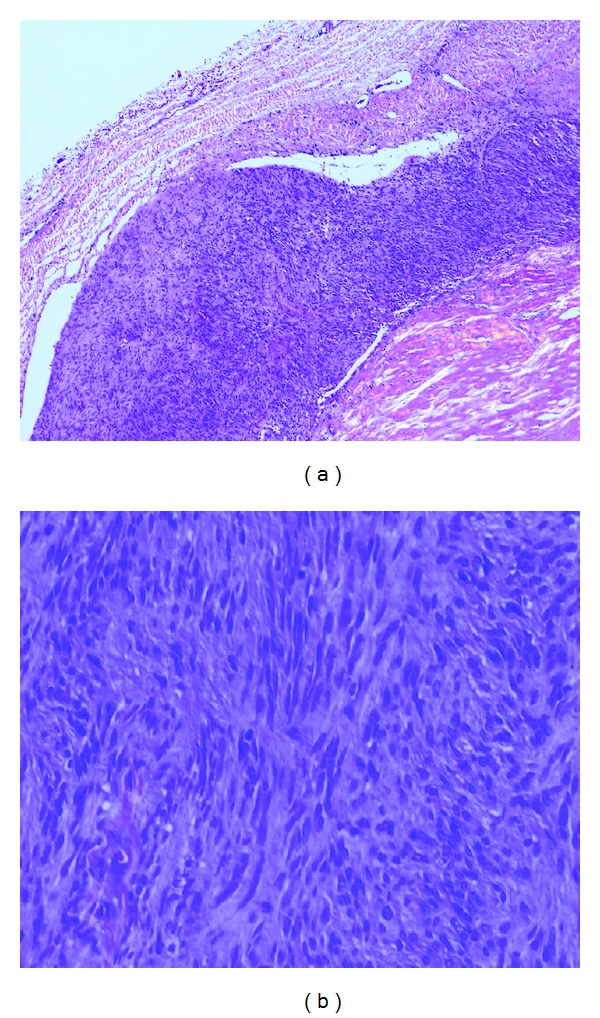
Photomicrograph reveals spindle-shaped tumor cells with “palisades,” which is compatible with the diagnosis of neurinoma (a) ×40; hematoxylin and eosin, (b) ×200; hematoxylin and eosin.

**Figure 4:**
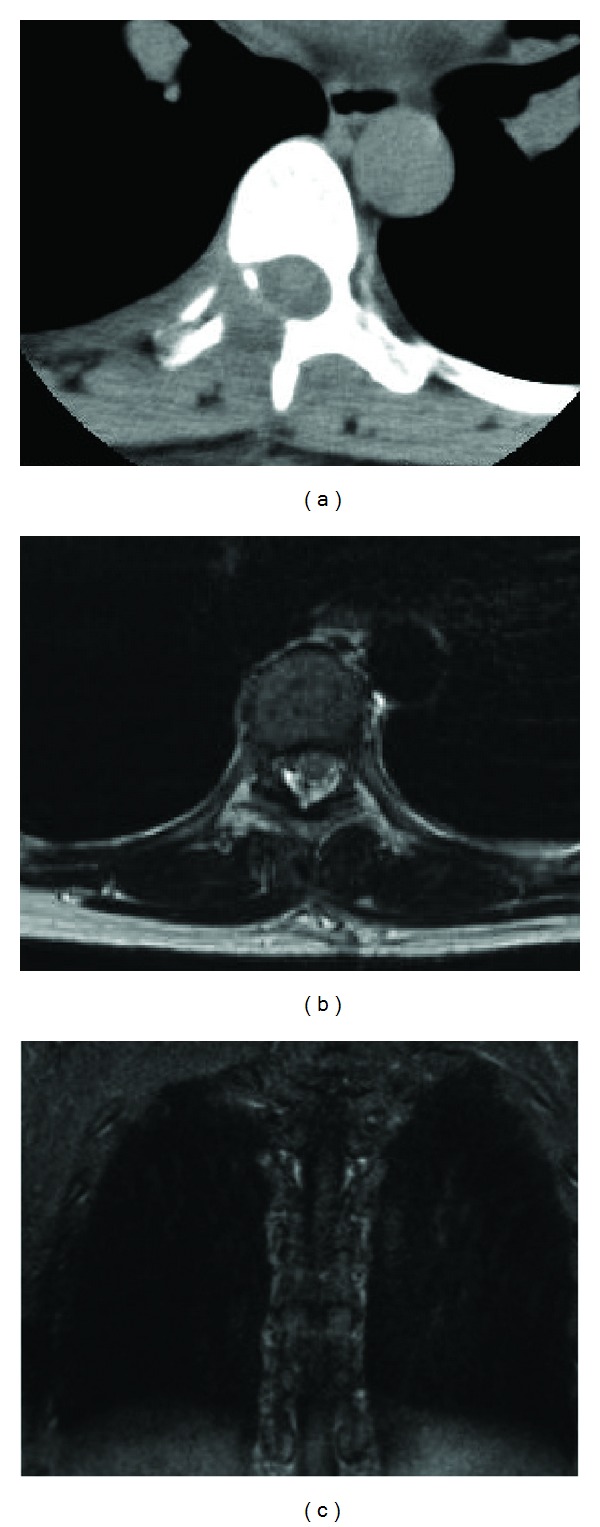
Postoperative hemilaminectomy of CT scan (a), axial (b), and coronal (c) views of T1-weighted MR images with gadolinium enhancement demonstrating complete removal of the tumor and decompression of the thoracic cord.

The patient's postoperative course was uneventful. She was discharged 8 days postoperatively without neurological deficits. Pathological examination demonstrated bundles of the spindle-shaped tumor cells arranged with typical feature of palisading containing degenerative tissue and deposition of hemosiderin (Figure 3). The histological diagnosis was neurinoma. Follow-up CT scan and MR imaging confirmed total removal of the tumor, and her symptoms had completely resolved (Figure 4).

## 3. Discussion

Dumbbell-shaped tumors are typically composed of an intraspinal and a paravertebral compartment. As previously reported, dumbbell-shaped tumors have been classified into four types [1, 14, 15]. For example, according to Eden's classification, dumbbell-shaped spinal tumors can be categorized based on location: (1) type 1, intra- and extradural; (2) type 2, intra- and extradural and paravertebral; (3) type 3, extradural and paravertebral; (4) type 4, foraminal and paravertebral. Type 3 tumors are the most common, and the present case was categorized as type 3.

Complete resection of the tumor impinging on the spinal cord is the most important objective in patients with a dumbbell-shaped thoracic cord tumor. Therefore, the posterior approach is usually used to remove the tumor. However, if the extraforaminal tumor component is large or the tumor is ventrally located, a combined posterolateral approach should be utilized. A lateral approach requires open thoracotomy or thoracoscopic surgery. In some ways, the thoracoscopic approach can be particularly advantageous when used for the removal of large intrathoracic tumors, because it offers clear visualization of the tumor and its adjacent vascular and mediastinal structures [5, 6].

Unilateral hemilaminectomy or partial hemilaminectomy has been advocated to minimize postoperative complications in vertebral stability. Hemilaminectomy provides adequate exposure to remove intradural-extramedullary and extradural tumors, and does not require cord manipulation [2, 16].

Endoscopic resection of the large neurogenic tumors usually requires widening of the stab wound. In the present case, the dumbbell-shaped tumors had already been separated from the thoracic dura and the nerve roots and had also been debulked via posterior route of hemilaminectomy. This facilitated en bloc resection of the tumor through the thoracic ports [2, 7–10, 13, 15].

Since the tumor in the intraspinal portion was tightly adherent to the dura, and, in the thoracic portions, was tightly adherent to the parietal pleural, the dumbbell-shaped neurinoma was managed using a two-staged approach: removal of the intraspinal portion followed by removal of the thoracoscopic portion. The intraspinal portion was resected first, after which the need for thoracoscopic resection was reevaluated and confirmed.

The benefits of thoracoscopic surgery over open thoracotomy include lower rates of various complications, including pulmonary complications, postoperative pain, intercostal neuralgias, shoulder girdle dysfunctions, and chronic pain syndromes [13, 17–20]. By avoiding rib retraction and muscle transection, thoracoscopic approaches reduce pulmonary morbidity and postoperative pain and are associated with shorter hospital stays than open procedure [13, 17, 20]. In addition to less morbidity, thoracoscopy preserves surgical efficacy for paraspinal neurogenic tumors. 

The goals of resection include obtaining tissue for diagnosis, decompression of thoracic cord, preventing tumor growth within the spinal canal, relieving mass effect within the chest, and preventing malignant transformation. Given the benign nature of neurogenic tumors, we do not believe that piecemeal resection poses a risk of malignant seeding to the patient.

In the present case, thoracoscopic surgery combined with hemilaminectomy was successfully used for the safe removal of the extraforaminal component. Fortunately, the risk of neurological deficit following resection of a nerve sheath tumor bearing the nerve root is low. Since the tumor can often be separated from the anterior dural root sleeve using blunt dissection with scissors, the nerve root can be preserved.

In conclusion, combined unilateral hemilaminectomy and thoracoscopic surgery is a good alternative for the resection of thoracic dumbbell-shaped extramedullary tumors.
